# Rumination and Loneliness Independently Predict Six-Month Later Depression Symptoms among Chinese Elderly in Nursing Homes

**DOI:** 10.1371/journal.pone.0137176

**Published:** 2015-09-03

**Authors:** Pei Gan, Yan Xie, Wenjie Duan, Qing Deng, Xiuli Yu

**Affiliations:** 1 Qinggang Senior Care Center, The First Affiliated Hospital of Chongqing Medical University, Chongqing, P. R. China; 2 Department of Applied Social Sciences, City University of Hong Kong, Hong Kong SAR, P. R. China; Federal University of Rio de Janeiro, BRAZIL

## Abstract

**Background:**

Previous studies conducted in Western countries independently demonstrated that loneliness and rumination are remarkable risk factors of depression among the elderly in both community and nursing homes. However, knowledge on the relationship between these three constructs among the elderly in Eastern countries is scarce. The current study aims to determine the relationship between loneliness, rumination, and depression among Chinese elderly in nursing homes.

**Methods:**

A total of 71 elderly participants with an average age of 82.49 years completed this six-month longitudinal study. Physical reports indicated that none of the participants were clinically depressed before the study. At Time 1, their loneliness and rumination were measured using UCLA-8 Loneliness Scale and Ruminative Responses Scale. Six months later, the participants completed the Center for Epidemiologic Studies Depression Scale to assess depressive symptoms (Time 2).

**Results:**

Multiple regression analysis revealed that both loneliness and rumination at Time 1 were the predictors of depression symptoms at Time 2 among the Chinese elderly in nursing homes. However, in the mediation analysis using PROCESS, the indirect effect between loneliness at Time 1 and depression symptoms at Time 2 was insignificant.

**Conclusions:**

Results suggest that previous loneliness and rumination thinking are predictors of future depression symptoms among the Chinese elderly in nursing homes. However, the insignificant mediation further suggests that the differences between loneliness and rumination should be explored in future studies. Findings have important implications for mental health professionals in nursing homes in China.

## Introduction

Depression has been a significant mental issue among the elderly around the world [1–3]; this mental condition usually causes poor health, emotional suffering, family disruption, mortality, and increased medical care costs [4–6]. A meta-analysis concluded that among the elderly with depression symptoms, only 33% remained healthy after two years, 33% became clinically depressed, and 21% died [7]. The majority of the elderly perennially stay in communities, and with the development of society, a growing number of them live in care homes [8]. However, as demonstrated in Western societies, the prevalence of depression differs between communities and nursing homes. For example, Barcelos-Ferreiraet al. [9]summarized the prevalence of depression in different communities, and found that nearly 3.80% to 15% of the elderly exhibit depressive symptoms. Although they obtain personal assistance and care in nursing homes, the prevalence was higher and more varied than that in communities, which ranged from 11% to 48% [10]. The high prevalence of depression symptoms in nursing homes implied the need to explore the risk factors and to clarify the mechanism of depression in nursing homes. An increased understanding of these issues will enable mental health professionals to reduce symptoms and facilitate positive aging of the elderly.

Previous studies identified loneliness as a remarkable risk factor of depression among the elderly. The relationships of the elderly often change with age and the transformation of society. Studies found that loneliness and lack of perceived social support are two relationship-related factors that accompany depression [11, 12]. Long-term reports further revealed that a harmonious intimate relationship was a protective factor of the low prevalence of depression among the elderly [13, 14]. After reaching 70 years old, loneliness that is positively related to negative emotion, dissatisfaction, and poor social support gradually increased with age [15–17]. In the early elderly period (65 to 80 years old), apathy and disability are equal contributors to depressive symptoms, whereas in the later elderly period (above 80 years old), apathy is the only significant predictor of depression [18]. However, longitudinal studies about loneliness and depression among the elderly are scarce, and considerable research should be conducted in the future [19]. All the aforementioned studies were conducted in the Western context, and the results may be inapplicable to China.

The Chinese Confucian culture may be prominent in the importance of loneliness in depression. Collective culture recognized relationship as a significant social norm and a virtue appreciated by society [20, 21]. A 14-year multiple-wave study (1993 to 2007) conducted in Taiwan, a typical collective culture region, revealed that increased loneliness and rapid industrialization and urbanization resulted in increased risk of depression among the elderly [22]. However, studies on the relationship between loneliness and depression in nursing homes in China are limited. Liu et al. [23] administrated a cross-sectional investigation, and found that social support partly mediated the relationship between loneliness and depression. Notably, no casual relationship was obtained. In modern China, nursing home is a promising choice for the elderly. The size of the family became smaller because of the impact of the “one-child policy.” The loneliness of the elderly will intensify after their children leave home or when a parent becomes widowed. Elderly people staying in a nursing home will obtain health care and peer support and will participate in social activities, which, in turn, decrease their sense of loneliness.

The association between loneliness and depression was established in various populations, but the mechanism underlying this relationship remains unclear. Rumination is a well-established trigger of depression among various populations [24]; rumination is repetitive thinking about the causes, consequences, and symptoms of negative affects [25]. Studies that involved older population have begun to explore methods to reduce their ruminant thinking, such as increasing one’s forgiveness [26] or improving one’s executive function [27]. Previous studies also indicated that a high level of hope usually results in less rumination among cancer survivors [28] and undergraduates [29]. However, studies that examined the role of rumination on loneliness—depression relationships among the elderly are few. Zawadzki et al. [30] conceptually distinguished loneliness and rumination, and further validated the full mediation role of rumination in the relationship between loneliness and depression among college students. No existing study has examined this hypothesis in the elderly. Thus, the present study hypothesizes that rumination can mediate the relationship between current loneliness and future depression symptoms among the elderly.

The present study aims to examine the relationship between loneliness, rumination, and depression symptoms among the elderly in nursing homes in China. In particular, the present study will examine (1) whether loneliness of the elderly in nursing homes in China can predict their future depressive symptoms, and (2) whether rumination can mediate the relationship between current loneliness and future depression symptoms among the elderly. The results of the current study will clarify the possible mechanism of loneliness in affecting depression symptoms and will facilitate the mental health service in nursing homes in China.

## Method

### Ethical Statement

All participants provided a written informed consent. The Institutional Review Board of the First Hospital Affiliated to Chongqing Medical University approved this study.

### Study Design

Convenience sampling method was adopted. All the elderly living in a nursing home in Chongqing City, which is located in southwest China in an area with moderate economic level, were invited to attend this investigation. The inclusion criteria are as follows: (1) individuals can read and write Chinese, (2)voluntarily participate in the survey, and (3) able to complete a self-reporting questionnaire package within 30 min in the dorm room. Participants with active physical and mental illnesses were excluded. One student who may provide guidance and who may answer any questions accompanied the investigation process. Participants need to complete the package at two different points to reduce cognitive load and fatigue.

### Procedures

Well-trained psychology students from Southwest University visited the dorm rooms of the elderly and helped them complete a questionnaire package in May 2014 (Time 1). Following the policy of the nursing home, each elderly person needed to be evaluated by the doctors and clinical psychologists before admission. Once any one of them was diagnosed with clinical depression disorder, they would be suggested to receive medical care before admission. The medical report indicated that none of the participants had been diagnosed with clinical depression. The elderly were required to report their gender, age, marital status, educational level, economic level, and medical history. Their levels of loneliness and rumination thinking were also assessed. Social volunteers visited them again after six months (i.e., November 2014, Time 2) to measure their depression symptoms using a self-report inventory.

### Participants

A total of 71elderly participants participated in the six-month project. The participants comprised 25 males (35.20%) and 46 females (64.80%). The mean age was 82.49± 5.86 years in the range of 66 to 94. Their average length of stay in nursing homes was 327.13 ± 238.23 days. The average monthly income of participants was CNY 3935.31± 1540.97. A total of 31elderly (43.70%) were married, 36 of them (50.7%) were widowed, and only 4 participants were separated or divorced. Approximately 29.6% elderly (n = 21) completed middle school education, 40.8% (n = 29) completed high school, and 29.6% (n = 21) had a bachelor’s degree. Approximately 74.6% of them (n = 53) regularly participated in physical exercise (e.g., Tai Chi, jogging, and walking), whereas only 43.70% of them (n = 31) regularly participated in social activities (e.g., chorus, dancing, and chess). The majority of the elderly had chronic illness including hypertension (n = 26), tumor (n = 16), cardiovascular disease (n = 15), and arthritis (n = 3).

### Measurements

#### UCLA-8 Loneliness Scale

UCLA Loneliness Scale [31] is a widely used inventory with good reliability and validity for measuring perceived loneliness among diverse populations. Hays and DiMatteo [32] developed a short version with eight items; their version was highly related to the original scale and was efficient for special samples (e.g., the elderly or the individuals with mental health issues). Previous study conducted among the elderly in China used this scale and supported its reliability and validity [33]. Participants were asked to rate each item on a four-point Likert scale, ranging from 1 (never) to 4 (always). Two of the eight items were reverse-coded. The higher mean score of the whole scale reflects the higher level of perceived loneliness. Cross-cultural studies revealed its stability across different countries and cultures [34, 35]. The Cronbach’s alpha coefficient in the current sample is .71.

#### Ruminative Responses Scale

Ruminative Responses Scale (RSS) is a 22-item questionnaire used to assess rumination response style [36]; this scale requires the participants to rate the extent of three kinds of responses as depressed mood; these responses include focusing on self, depressive symptoms, and the causes and consequences of the mood. Treynoret al. [25] re-examined the factor structure of RRS and recommended using a two-factor structure to reduce confusion on the symptoms of depression reflected by RRS. In the current study, a 10-item short version was adopted to measure two sub-factors of rumination, namely, brooding (5 items) and reflection (5 items). The respondents were asked to rate the frequency of each item on a four-point Likert scale. High mean scores of the whole scale indicates high level of rumination response style. The RRS has been applied in the elderly population in previous studies [26]. The Cronbach’s alpha in the present sample is .77.

#### Center for Epidemiologic Studies Depression Scale

The Center for Epidemiologic Studies Depression (CES-D) scale is a 20-item scale and is commonly used to self-report test to measure depressive symptoms in general population [37]. Participants were required to report the frequency of depression symptoms over the last week on a four-point Likert scale, ranging from 0 (rarely or none of the time) to 3 (most or all of the time). Four items (4, 8, 12,16) are reverse-scored. High mean scores of the total scale reflects high level of severity of depression symptoms. Radloff [37]demonstrated that the CES-D is a reliable and valid measure of depressive symptoms in various populations, including the Chinese older population [38]. The Cronbach’s alpha of CES-D in the current sample is .77.

### Data Analysis Plan

SPSS 22.0 was used to analyze the data. The demographic profile of the target sample was first obtained via descriptive and frequency analysis. The mean and standard deviation scores of loneliness, rumination, and depression symptoms were calculated. The Pearson correlation coefficient was used to measure the correlations between loneliness and rumination at Time 1 and the depression symptoms at Time 2. Hierarchical regression analysis was then conducted to reveal the contributions of loneliness and rumination to future symptoms of depression. The scores of depression were set as dependent variables. Correlations between demographic variables and psychological variables were examined to determine whether any of the demographic variables should be controlled in the regression. The demographic variables were controlled and entered into the first step. The scores of loneliness were entered in the second step and the scores for rumination were entered in the third step. Finally, the mediation effect was examined using Model 4 in PROCESS [39] program. Loneliness was set as predictor (X), depression was set as outcome (Y), and rumination was set as mediator (M).

## Results

The mean scores, standard deviation, and correlation coefficients are shown in Table 1. Correlation analysis shows that loneliness at Time 1was significantly related to rumination at Time 1(*r* = .25, *p*< .05) and depression at Time 2(*r* = .30, *p*< .001); rumination at Time 1was significantly related to depression at Time 2(*r* = .30, *p*< .001).

**Table 1:** Descriptive and Pearson Correlation Analysis. (N = 71).

	Range	*Mean*	*SD*	2	3
**1 Loneliness**	1.00~2.63	1.53	0.40	.25*	.30**
**2 Rumination**	1.00~2.30	1.48	0.35	-	.30*
**3 Depression**	0.00~1.90	0.87	0.35	-	-

Note.

*SD* = Standard Deviation.

* *p*< .05;

** *p*< .01.

Among the demographic variables, only illness history (*r* = .24, *p* = .048) was significantly related to rumination. Thus, the illness history was regarded as controlled variable in regression analysis. After controlling for illness history, the results of the hierarchical regression analysis in Table 2 demonstrated that only rumination was a significant predictor of future depression symptoms among the elderly in nursing homes in China. Loneliness at Time 1 could not predict depression symptoms at Time 2.

**Table 2:** Hierarchical Regression Analysis on the Depression Symptoms. (N = 71).

Dependent Variable: Depression	Step 1		Step 2		Step 3	
	*Beta*	*t*	*Beta*	*t*	*Beta*	*t*
**Illness History**	.052	0.431	.038	.333	-.016	-.135
**Loneliness**	-	-	.302	2.617*	.245	2.099*
**Rumination**	-	-	-	-	.286	2.007*
**R** ^**2**^	.003		.094		.145	
***F***	.186		3.525*		3.797*	
**ΔR** ^**2**^	-		.091		.107	

* *p*< .05.

Finally, the mediation effect was examined. The direct and indirect effects of loneliness and rumination on depression was calculated by the SPSS macro developed by Hayes [39], which adopted the bootstrapping strategy. In the present study, each test was resampled 5,000 times and accelerated at 90% confidence interval. Illness history was set as covariates. Table 3 shows that the overall models were significant.

**Table 3:** Mediation Effect Analysis Based on PROCESS. (N = 71).

	*R* ^*2*^	*F*	*Coeff*.	*SE*	*t*	*p*	LLCI	ULCI
**Outcome: Rumination**	0.113	4.319*						
** Loneliness**			0.210	0.101	2.093	0.040	0.010	0.411
** Illness History**			0.056	0.029	1.968	0.053	-0.001	0.113
**Outcome: Depression**	0.145	3.797*						
** Rumination**			0.242	0.121	2.007	0.049	0.001	0.483
** Loneliness**			0.216	0.103	2.099	0.040	0.011	0.422
** Illness History**			-0.004	0.029	-0.135	0.893	-0.062	0.054

Note.

*Coeff*. = Unstandardized Coefficients;

*SE* = Standard Error;

LLCI = Lower Limit Confidence Intervals;

ULCI = UpperLimit Confidence Intervals.

* *p*< .05.

**Fig 1:**
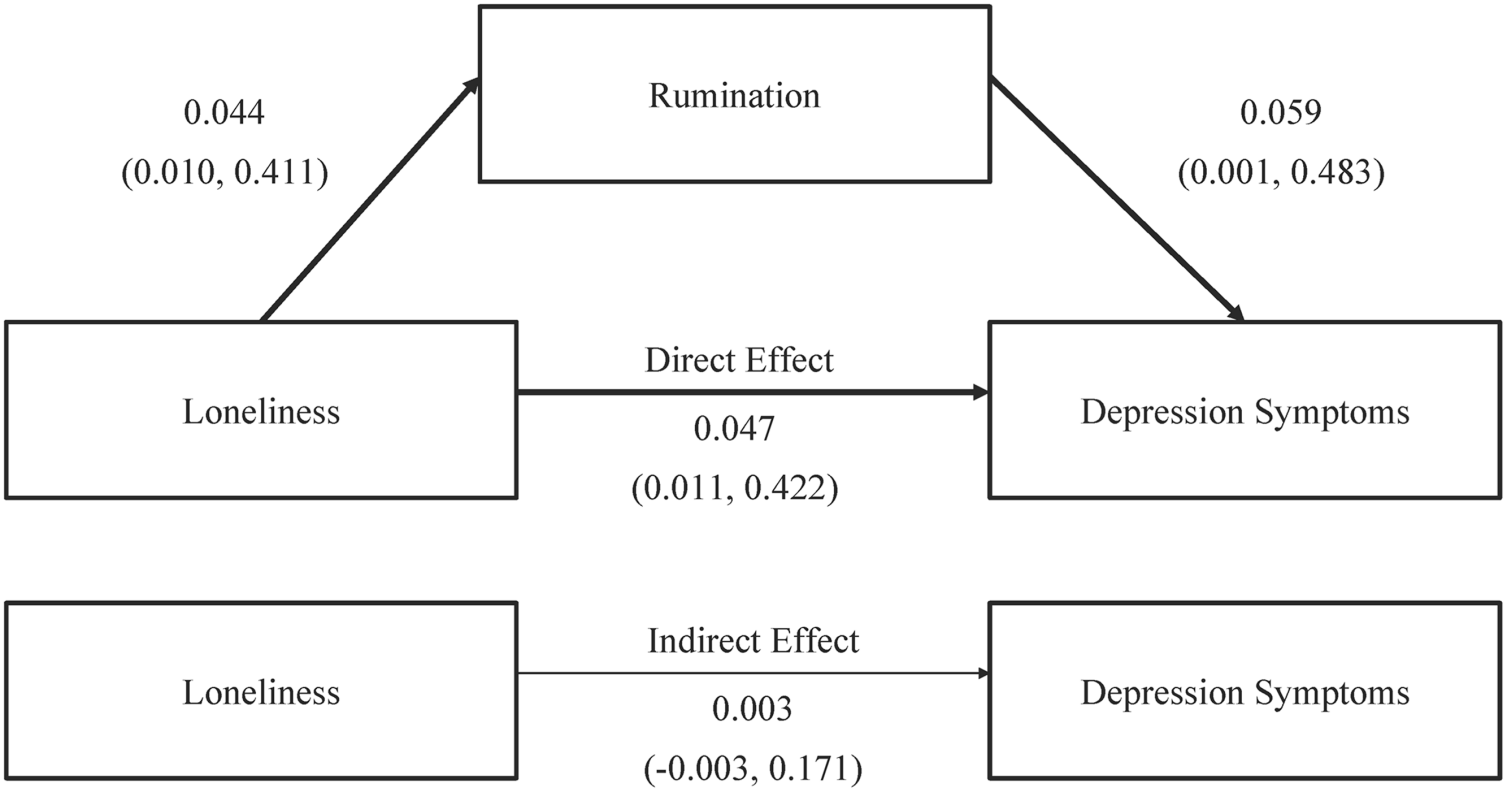
The Estimation of the Direct and Indirect Effect of Loneliness in Affecting Rumination and Depression among the Elderly in Nursing Homes of China. (N = 71). Note. Brackets indicated the bias-corrected and accelerated 90% confidence interval. Bolded lines indicate the significant effects. Illness history is controlled as covariates.

Before entering rumination in the equation, only loneliness (*t* = 2.617, *p*< 0.05) significantly predicted depression, whereas both loneliness (*t* = 2.099, *p*< 0.05) and rumination (*t* = 2.007, *p*< 0.05) became the significant predictors of depression after entering rumination. Fig 1 shows the estimation of the direct and indirect effects of loneliness on rumination and depression. However, the results of mediation analysis in Table 3 and Fig 1 indicate that rumination cannot mediate the relationship between loneliness and depression. In another words, the indirect effect reflected by Fig 1 was insignificant.

## Discussion

The study investigated the relationship between loneliness, rumination, and depression symptoms among Chinese elderly in nursing homes with an average age of 82.49 years. This study finds that both loneliness and rumination can predict future depressive symptoms. However, rumination cannot mediate the relationship between loneliness and depression.

Previous studies indicated that loneliness is an important risk factor of negative emotions and cognitive states, including depression and anxiety, and the synergistic interactions of loneliness can further diminish the well being of the elderly [31, 40, 41]. Other studies adopted the sample with an average age of 65 years, and reported that high levels of loneliness are associated with high levels of depression [15, 41, 42]. The present study similarly finds a direct predictive relationship between loneliness and depression. This finding has important implications for care workers. Certain cross-sectional studies found that social support negatively mediated the relationship between loneliness and depression among the elderly, which suggests that a high level of perceived social support could result in less loneliness and depressive symptoms [23, 43]. The participants in the present study lived in nursing homes and obtained social support from different pathways in daily life, including registered care workers, health consultants, and their peers. The perceived social supports of the elderly were higher than of those who live in communities, which, in turn, resulted in low level of loneliness and depression. Future longitudinal studies should be conducted to explore whether loneliness and the perceived social support of the elderly will change after staying in nursing homes. However, select studies demonstrated that the higher age has a protective effect on the depression symptoms of the elderly in care homes [10], which means that loneliness increased the difficulty in predicting depression symptoms among the elderly with higher age. Therefore, more studies should be conducted in future to examine whether age is an important factor in the development of depression symptoms.

An interesting finding in the current study is the insignificant mediation of rumination on the relationship between loneliness and depressive symptoms after six months. This result implies that the concepts of loneliness and rumination should be clearly distinguished. As discussed in the Introduction, a few researchers have begun to explore the conceptual relationship between loneliness and rumination [30], as well as examined their relationships among college students. Studies that examined the relationship between loneliness, rumination, and depression among the elderly are limited [30]. For example, Vanhalstet al. [44] studied the relationship between these constructs. However, they used a college sample and demonstrated that uncontrolled ruminative thoughts mediated and moderated the relationship between loneliness and depressive symptoms. These results implied that the conceptual relationship between loneliness and rumination among the elderly was different from that between loneliness and rumination among college students. More studies are needed to explore this important issue.

The results also provide two important implications for mental health professionals in nursing homes. First, social support system should be established in nursing homes by developing diverse sports, culture, and social activities to help the elderly reduce their loneliness and increase their perceived social support. Mental health professionals in nursing homes should also actively devote to changing the rumination of the elderly by adopting positive psychological intervention and positive psychological therapies, such as CBT and MBCT. Such an action can prevent these elderly from falling into the pitfall of rumination because of loneliness. Hence, future research should pay attention to the establishment of a social support system and a psychological intervention program, which can be used to examine the efficacy of relieving symptoms of elderly depression. Notably, the present study is an exploratory study and further studies are needed with larger sample sizes where baseline depressive symptoms are gathered. Therefore, the abovementioned implications must be re-examined.

A few limitations of the present study should be mentioned. First, the sample size of the current study is too small, which makes identifying the aged and educated elderly who completed long questionnaires difficult. Future studies may adopt a large sample to replicate the present study. Second, some other rated measurements or performance-based tests should be used in future studies to replace the current self-report measurements. In addition, the measurements that used in the current study (i.e., RRS) need to be validated among the Chinese elderly. Better depression assessment tools should be adopted, such as Geriatric Depression Scale and Cornell Scale for Dementia. Third, the results obtained in the current study cannot be recognized as clinical study results. Future studies should be conducted in nursing homes and communities using samples with similar characteristics to determine if the environmental factors of nursing homes in China are proactive to depression. Finally, this study cannot be recognized as a complete longitudinal study because no baseline measurement of depressive symptoms was available. No participant was diagnosed with clinical depression, but this case does not mean the elderly did not have depressive symptoms. Thus, more rigorous longitudinal design is necessary to validate the current results.

## Supporting Information

S1 Dataset (SAV)
